# Medical Convention of Ohio

**Published:** 1834

**Authors:** 


### Art. II.—Medical Convention of Ohio.

Pursuant to a circular letter, addressed to "all Scientific Practitioners of Medicine and Surgery in the State of Ohio," recommending a General Medical Convention to be held in the city of Columbus, on the 5th day of January, A. D. 1835, for the purposes therein mentioned and expressed, the following persons assembled in the basement room of the Presbyterian church, in said city, on the day and year aforesaid, at 10 o'clock, A. M., to wit:

Doctor Thomas Earl, of the county of *Portage*.

Doctors Robt. McNiell, M. Z. Kreider, —— Anderson, John Dougherty, and A. A. Frisbee, of the county of *Fairfield.*

Doctors Samuel Parsons, W. M. Awl, P. Sisson, M. B. Wright, Robt. Thompson, N. Miller, —— Robinson, C. H. Wetmore, Geo. Snow, Kingsly Ray, J. Clark, B. F. Gard, and N. Boalse, of the county of *Franklin.*

Doctors Peter Allen, and Jared Potter Kirtland, of the county of *Trumbull.*

Doctor G. W. Woolley, of the county of *Huron.*

Doctor John Cook, of the county of *Jefferson.*

Doctor Robert Houston, of the county of *Clarke.*

Doctors James Ewing, H. M. Thrall, William Bancroft, Geo. W. Landon, —— Tracy, and —— Scott, of the county of *Licking.*

Doctors D. W. Rhodes, A. H. Brown, L. P. Blocksom, James Helmick, Wm. F. Coleman, John A. Turner, Robt. Safford, and Geo. W. Moorhead, of the county of *Muskingum.*

Doctor P. B. Johnson, of the county of *Morgan.*

Doctors Wm. Trevitt, and Philip Harvey, of the county of *Perry.*

Doctors J. B. Thompson, and I. O'Ferrall, of county *Guernsey.*

Doctor J. M. Dillon, of the county of *Monroe.*

Doctor Joshua Martin, of the county of *Greene.*

Doctor Edwin W. Smith, of the county of *Montgomery.*

Doctor J. Parker, of the county of *Preble.*

Doctors George Keefer, Asa Coleman, John O'Ferrall, and —— Hamilton, ofthe county of *Miami.*

Doctors Daniel Drake, Thos. D. Mitchell, John Eberle, Robert P Simmons, L. C. Rives, Silas Reed, Charles R. Cooper, and William Mount, of the county of *Hamilton.*

Doctor L. A. Hendrick, and Wm. Doane, of the county of *Clermont.*

Doctors Wm. M. Charters, Wm. Harvey, and Isaac Fisher, of the county of *Warren.*

Doctor James Moore, of the county of *Ross.*

Doctor Jacob Kirby, of the county of *Highland.*

Doctor Jacob Dewees, of the county of *Putnam.*

Doctors Thomas H. Gibson, and B. S. Olds, of the county of *Pickaway.*

Doctors C. Ankhelen, and —— Tolland, of the county of *Madison.*

Doctor Carnell, of the county of *Delaware.*

The meeting was organized by calling Doctor McNiell to the Chair, and appointing Doctors Awl and Kreider Secretaries pro tempore.

Whereupon, the circular of last June, calling the Convention, was read by its author, Dr. Wm. M. Awl, as follows:

*To all Scientific Practitioners of Medicine and Surgery in the State of Ohio.*

The undersigned, uniting in sentiment and feeling with that portion of the Profession who view, with pain, the great depression of character—want of harmony and concentration of useful action, which unhappily prevail in the Medical Science—acknowledging, also, a proper responsibility for the advancement of correct principles, the promotion of public benevolence, and the common welfare of society, —are induced most respectfully to recommend and consent to support a call for the assemblage of a General Medical Convention, to be holden in the city of Columbus, on Monday, the 5th of January, A. D. 1835.

The grand design is to organize for practical utility, the whole scientific medical power of the State. All regular scientific Practitioners of Medicine and Surgery, either of city, village, or country, who are disposed to advance the honor and dignity of the Profession;—every one who has a heart in the cause of science, and is ready to unite with the great and good of the age, in elevating the moral and scientific character and talent of the great and extending West, is cordially invited, and expected to come and record his name in this Convention.

*The regulation of professional etiquette—The construction of independent Medical Societies—The support of a periodical Journal of Practical Medicine—The erection and location of public Asylums, for the reception of Lunatics and the instruction of the Blind—The promotion of the Temperance cause—The regulation of Vaccination—The convenient supply of the Leech:—*

And many other subjects will, perhaps, claim the attention of the Convention. But as the whole proceeding should be an independent and voluntary offering for the common good, all are expected to be unpledged, and none should come entirely unprepared.

On motion of Dr. R. Thompson,

*Resolved,* That a Committee of six be appointed, to nominate the officers of the Convention.

Whereupon, the Chair appointed Doctors Robert Thompson, Saml. Parsons, D. W. Rhodes, Silas Reed, M. B. Wright, and J. A. Parker, said committee.

Doctor Wetmore offered the following resolution:

*Resolved,* That a Committee of three persons be appointed, to inquire into the rights of individuals to membership in this Convention, —which was, on motion of Dr. Rhodes, indefinitely postponed.

On motion of Dr. Mount,

*Resolved,* That the vote so taken for indefinite postponement be reconsidered, and the aforesaid resolution, offered by Dr. Wetmore, was adopted unanimously. Whereupon the Chair appointed Doctors Cooper, Eberle, and Rives, said committee.

The Chair announced an invitation from Charles Anthony, Esq., one of the Directors of the Ohio Penitentiary, to visit that institution, at 2 o'clock, P. M. of this day, which communication was ordered to be filed, and the invitation accepted.

Dr. Thompson, from the committee appointed to nominate officers, reported the names of the following gentlemen, as officers of the Convention, to wit:

| | | |
|---|---|---|
| *For President,* | PETER ALLEN, of Trumbull Co. | |
| 1st *Vice President,* | L. C. Rives, | Hamilton. |
| 2d " | Robt. McNiell, | Fairfield. |
| 3d " | J. A. Parker, | Preble. |
| 4th " | D. W. Rhodes, | Muskingum. |
| 5th " | J. P. Kirtland, | Trumbull. |
| 6th " | Jacob Kirby, | Highland, |

*Corresponding Secretary,* Wm. M. Awl, Franklin.
*Recording Secretary,* M. Z. Kreider, Fairfield.
*Treasurer,* M. B. Wright, Franklin.

Which Report was, on motion, adopted.

Dr. Cooper, from the committee on the subject of the 'rights of membership,' reported as follows, to wit:

"Your committee, appointed to inquire into the rights of individuals to membership, in this Convention, *Report*—That the obligations the Convention owe to the profession, require it of them that in order that an individual shall be entitled to a seat in this body, he shall have been a regular student of medicine, under the direction of a respectable and qualified physician, and that all disciples of 'Botanic' or 'Thompsonian' systems of practice, be excluded from all participation in the deliberations of this Convention." Which report was agreed to.

On motion of Dr. Drake,

*Resolved*, That the committees on all the great subjects introduced for discussion into this Convention, shall be appointed by the President, assisted by the Vice Presidents.

On motion of Dr. Drake,

*Resolved*, That a committee of five members be appointed to draft a report and resolutions on the subject of Commercial Hospitals in the Valleys of the Mississippi and the Lakes, to be erected by the General Government Whereupon, Doctors Drake, Brown, Earl, Cook, and Kirtland, were appointed said committee.

On motion of Dr. Kreider,

*Resolved*, That a committee of three members be appointed to report upon the expediency of memorializing the Legislature on the subject of legalizing the study of Anatomy. Whereupon, Doctors Kreider, Mitchell, and Cooper, were appointed said committee.

On motion of Dr. Rives,

*Resolved*, That a committee of four be appointed, to inquire into and report upon the means of improving the state of Medical Education in Ohio. Whereupon, Doctors Rives, Drake. Awl, and Reed, were appointed said committee.

On motion of Dr. Coleman,

*Resolved*, That a committee of three members be appointed to prepare a report on the subject of Vaccination. Whereupon, Doctors Coleman, Hamilton, and Woolley were appointed said committee.

On motion of Dr. R. Thompson,

*Resolved*, That a committee of five be appointed to inquire into the expediency of memorializing the Legislature of Ohio upon the subject of a Lunatic Asylum, and make report thereon. Whereupon, Doctors Thompson, Smith, Awl, Eberle, and Mitchell, were appointed said committee.

On motion of Dr. Drake,

*Resolved*, That a committee of three members be appointed to prepare a report and resolutions on the subject of a School for the Education of the Blind, to be established by the Legislature of Ohio, in the city of Columbus. Whereupon, Doctors Drake, Rhodes, and Parsons were appointed said committee.

On motion of Dr. McNeill,

*Resolved*, That a committee of three be appointed, to inquire into the expediency of memorializing the Legislature on the subject of a law regulating the practice of medicine. Whereupon, Doctors McNiell, Parsons, and Martin, were appointed said committee.

On motion of Dr. Wetmore,

*Resolved*, That a committee of three be appointed to prepare a report to be presented to this Society, on the subject of Intemperance, in conformity with the views of the circular. Whereupon, Doctors Wetmore, Awl, and Kreider, were appointed said committee.

On motion of Dr. Kirtland,

*Resolved*, That a committee of three members be appointed to report to this meeting a system of medical Ethics. Whereupon, Doctors Kirtland, Eberle, and Reed, were appointed said committee.

On motion of Dr. Awl,

*Resolved*, That a committee of three be appointed, to report on the support of a practical Journal of Medicine, suited to the wants of the profession in the West. Whereupon, Doctors Awl, Eberle, and McNiell, were appointed said committeee.

On motion of Dr. Wright,

*Resolved*, That a committee of three be appointed to report to this meeting upon the convenient supply of the *Leech*. Whereupon, Doctors Wright, Awl, and Cook, were appointed said committee.

The Chair then read a communication from a committee of the Ohio State Temperance Society, requesting the Convention to appoint a member thereof to deliver an Address before that Society, at such time as shall be convenient. Whereupon, Doctor Mitchell was selected by the Chair to perform that duty.

On motion, the Convention adjourned until 9 o'clock, A. M. to-morrow, to meet in the court room of the Circuit Court.

*Court Room, Jan.* 6, 1835,—9 *o'clock, A. M.*

The Convention met pursuant to adjournment. The President in the Chair. The minutes of yesterday were read and adopted.

Dr. Cooper submitted the following:

"That the diffusion of knowledge on the instruction of the Blind, is, in the estimation of this Convention, of paramount importance—that the great, the good, and the philanthropic of the age, have enlisted in the promotion of this noble cause—and that we are called upon by every feeling which dignifies our nature, by every consideration worthy of the mind of man, and by every benevolent impulse of the heart, to lend our aid and influence in the furtherance of this great object—therefore,

"*Resolved*, That Dr. Daniel Drake be requested to deliver an address on the subject of the Instruction of the Blind, to-morrow (Wednesday) evening, at half past 6 o'clock, and that both Houses of the Legislature of Ohio be respectfully invited to attend." Which resolution was adopted.

Dr. DRAKE, from the committee to whom was referred the subject of Western Commercial Hospitals, made the following

### REPORT:

The necessity for Hospitals in the Valleys of the Mississippi and the Lakes, for the relief of those engaged in our commerce, and also, of travellers, had been perceived from the infancy of the Western settlements; but it was left for a benevolent physician, of the State of Missouri, Dr. CORNELIUS CAMPBELL, of St. Louis, to set on foot the project of urging the General Government to establish them. The Committee have given to this subject the serious attention which its importance demands, and have come, unanimously, to the conclusion, that it should be earnestly and respectfully pressed on the attention of Congress.

In presenting their views to the Convention, they begin, first, by a reference to the geographical extent of the region, which is immediately interested in the object. Its area is greater than that of the remainder of the Union—embracing large and populous portions of New York, Pennsylvania and Virginia, a part of Alabama, most of Mississippi, and the whole of Louisiana, Tennessee, Kentucky, Ohio, Indiana, Illinois, and Missouri, with the Territories of Arkansas and Michigan. Regarding the last, as entitled by its population to admission into the Union, the number of States embraced in the region for which the Hospitals are intended, is equal to the original thirteen. Their population is already more than one-third of that of the whole Union, and is rapidly increasing. The lake navigation, carried on by means of schooners and steamboats, extends from Buffaloe, in New York, at the eastern end of lake Erie, to Chicago, on the south western shore of lake Michigan, in Illinois, a distance of more than 1000 miles. The rivers of the Lakes and of the Missisippi Valley, which are already navigable with flats, keels, and steamboats, may be estimated at 6,000—making an aggregate of 7,000 miles.

Secondly. The extent of this navigation necessarily includes a great variety of latitudes, with voyages so distant, as to subject those who are engaged in our commerce, to the influence of different, and to them unhealthy climates; while the banks of all the navigable waters, like those of every new country, send forth insalubrious exhalations. From the combined action of these causes, no inland navigation, in the temperate zone is, perhaps, more unhealthy. The ascending steamboat voyage, every year, brings up the Mississippi and its tributaries, a great number of deck passengers, chiefly the sons of farmers, of whom many die on board; but the number that perish in the long and sickly descending voyages of the flat bottomed boats, which often depart from the upper waters in summer and early autumn, when they are low and their shores unhealthy, must be still greater. Nothing is more common than for two out of the five hands, who generally manage one of these boats, to die; and it has even happened, that the whole have perished, and the boat with its cargo been left deserted, to be lost.

Third. Your committee have found it impossible to make an accurate estimate of the number of persons engaged in the lake and river trade. The Mississippi and its tributary streams, have, at the present time, about 250 steamboats in commission. The average number of persons engaged in navigating them, as your committee have been informed by experienced steamboat commanders, may be stated at 22, giving a total of 5,500. The number of flat bottomed boats which float down the Mississippi is quite unknown. Several years ago it was ascertained that 10,000 arrived in a single year at New Orleans. At Memphis, in Tennessee, below the mouth of the Ohio, it was estimated, that in 1833, 5,000 passed down. Your committee, believing that the multiplication of steamboats has diminished the number of flats, incline to the latter estimate, and will assume 6,000, which, at an average of four and a half hands for each boat, will give 27,000. In addition, the coal, salt, and lumber trade of the Ohio, and the fur trade of the Missouri, may give 7,500, making a sum total for the trade on the southern or Gulf declivity of the West, of 40,000. The number of steamboats on the lakes is about 35, averaging, from their size, 28 hands, amounting in round numbers to 1,000. The schooners, sloops and brigs, on the same waters, are not less than 250, having six hands to each, and giving 1,500; to which may be added, 500 for the Erie and Ohio canal, giving an aggregate for the lakes of 3,000; which, added to the previous sum, gives a grand total of 43,000. It appears, from the foreign and coasting marine tonnage of the United States, in 1832, that allowing a seamen for every 20 tons, the whole number was about 63,000. Thus the number of persons employed in the commerce of the West, is more than two-thirds of the number engaged in the maritime commerce of the Union.

The boatmen, are, however, but a part, and not the larger part, of those who navigate the Western waters. The commercial and social relations of the North and the South, and the practice of escaping from the latter to the former at the opening of summer, throngs our steamboats with passengers, many of whom fall sick and die on the voyage.

Fourth. The necessity of numerous hospitals for the reception of the sick, and those who suffer from steamboat explosions and other casualties, cannot fail to be perceived and admitted, by all who may be induced to view the subject as your committee have seen it. From the absence of such hospitals, at short intervals, many valuable lives have been lost, that might have been preserved, if *timely* aid had been administered. This is sometimes done, by the skill and humanity of steamboat commanders; but the flat boats are, generally, unprovided with the requisite medicines, and on board of both, it is not uncommon for persons to sicken and die, without medical assistance; the boat still keeping on her way, and only stopping to deposit the remains of the deceased, in some new village, or underneath the trees of the forest.

Fifth. Your committee are not prepared to speak definitively of the number and locality of the hospitals, which an extension of the trade of the West will ultimately require; nor even to point out with precision the points, where, at this time, they are most needed; but they venture to indicate the following, as the places where they are now wanted: New Orleans, Natchez, Memphis, St. Louis, Louisville, Cincinnati, Pittsburgh, Buffaloe, Cleaveland, and Detroit; to which they will add, Chico, or some other point near the mouth of the Arkansas, Trinity, or a spot near the junction of the Ohio and Mississippi; Evansville or some point still less remote from the mouth of the Wabash; and Parkersburg, or Guyandotte, in Western Virginia. With such, or even a less number, no sick person, on board a steamboat or schooner would suffer longer than one or two days before he could be lodged in a hospital, where the early application of medicine might preserve his life, and permit him to prosecute his voyage in a subsequent boat; and those who descend our rivers in flat boats would not, in general, be more than two or three days, in any part of their exposed and tedious voyage, without being able to command the requisite medical advice, whereby many valuable lives would be saved, and the prosperity of our commerce greatly promoted.

Sixth. The location, erection and support of these hospitals, are not likely to be accomplished by the different States; for justice requires, that the advantages they would afford should be reciprocally enjoyed, and, therefore, that they should not only be erected at the same time, but in such places as would combine the whole into one great system of national charity; neither of which could be accomplished without such negotiations between the States interested, as are not contemplated in the Federal Constitution.

Seventh. Your committee, therefore, are of opinion, that the work properly and constitutionally belongs to the General Government. That it is strictly national. That the trade which it is designed to cherish, is not only a part of the coasting, but the foreign trade of the United States; and that it is entitled to the same fostering care of the Federal Government, with that of the old Atlantic States, for which similar provision has been already made. Your committee, moreover, regard the commerce of the West as a nursery of seamen; for in the event of a maritime war, the boatmen of the lakes and the Mississippi would make both willing and able sailors.

Eighth. Still further, these hospitals would often be found serviceable in the transportation of our troops under the peace establishment; and in case of a continental war with a foreign power, they would offer very great advantages to our armies, whether stationed on the Lakes or the Gulph, or marching to or from either.

Ninth. Without intending to forestall the wisdom of Congress, to whom your committee hope this subject will be presented by the Convention, they would suggest that there are two obvious sources of the ways and means, for the accomplishment of this important object. First, the per centage of 20 cents a month while in actual service, on steamboat and ship hands, similar to that on the ocean. The sum that might annually be raised at this rate, would, if 8,500 are employed half the year, amount to 5,100 dollars. But this sum would not be sufficient to support hospitals for the steamboat and schooner sailors alone, much less to embrace the far greater number that are engaged in the management of flat bottomed boats; which, fitting out and departing from a countless number of places, and each one designed for a single voyage only, are not and never will be generally registered in any port of entry. Secondly, the public lands lie chiefly in the basins of the Lakes and the Mississippi, and are augmented in value, by whatever promotes the prosperity of those who have already purchased. Apart, then, from all considerations of justice to the West, your committee are of opinion, that those who hold the national domain in trust, would be in the direct execution of that trust, having the value of the remainder in view, were they to pledge a part of the proceeds of the land sales to this great object. But your committee, as citizens of the West, would urge upon Congress, that appropriations of the public money should, as far as practicable, be equal over the nation; that hitherto their elder brethren of the sea-board have been favored with far greater disbursements than they; and that in erecting and permanently endowing, the proposed system of Commercial Hospitals, they would but do that, which the Western States may of right claim, as portions of the Union.

Tenth. In several places, as New Orleans, Natchez, Memphis, Louisville and Cincinnati, hospitals have already been erected by the States. These might, by arrangement, be made to receive the class of persons, for whom the general government would provide, those establishments receiving an annual appropriation. But in the other places, it would be necessary to erect and furnish hospital edifices; which in most cases might, perhaps, be done in connection with the States. When erected by the Federal Government alone, and designed merely for boatmen, they need not be large, as the number of patients at no time would be great. As to the *relative* endowment of these hospitals, it must be according to the judgment of Congress, when directed by the facts which indicate the relative number of the sick which they might respectively admit into their wards. New Orleans should obviously receive a larger appropriation than Natchez, and Louisville more than Pittsburgh; but your committee do not venture on these details any further, than to express an opinion in favor of unequal endowments, as required by inequality in the responsibilities of different places.

Imperfect as this report necessarily is, from the short time allowed for its preparation, not less than the novelty of the subject, and the difficulty of procuring correct data, your committee indulge the hope, that it will be found sufficient to ensure the co-operation of the Convention, in the great work of national beneficence, which it is designed to promote. It is due to the character of our profession, that we should zealously aid in every work of public charity, that has for its object the mitigation of human suffering and the preservation of life; and the physicians of the immense range of new and rising States, immediately interested in this great object, especially owe it to Western society, to exert themselves in its promotion. Your committee have confidence in the enlightened benevolence of their brethren, and do not hesitate to expect a hearty and efficient co-operation.

Your committee beg leave to propose the following resolutions:

1. *Resolved*, That a copy of this report and resolutions be transmitted to our General Assembly, through his Excellency the Governor, to the end, that if the objects set forth should meet the approbation of that honorable body, it may present a memorial on the subject to Congress.

2. That printed copies of the same be transmitted to the

President of the United States, the Vice President, Members of Congress, and the Heads of Departments.

3. That printed copies be forwarded to the Governors of all the States and Territories of the Union.

4. That 500 additional copies be printed for distribution by the members of the Convention, throughout the West.

5. That it be published in the newspapers of Columbus.

6. That the Corresponding Secretary be charged with these distributions. DAN. DRAKE, *Chairman.*

*Jan.* 6, 1835.

Which Report was agreed to. Whereupon it was

*Resolved,* That Dr. Drake be authorised to receive one hundred copies of the above Report, extra.

Dr. McNiell, from the Committee on Medical Laws, reported as follows:

The Committee appointed under the following resolution—"Resolved that a committee of three be appointed, to enquire into the expediency of memorializing the Legislature on the subject of a law regulating the practice of Medicine"—have taken the same under consideration, and after due deliberation, unanimously concur in opinion that any effort of the kind at this time, would be premature and inexpedient.

Which Report was adopted, and a motion to reconsider was lost.

The Chair read an invitation to the Convention, from Mr. H. H. Hubbell, Principal of the Deaf and Dumb Asylum, to visit that institution; which was ordered to be filed, and the invitation therein contained, was accepted.

On motion of Dr. Gard,

*Resolved,* That a Committee of three members be appointed, to report a memorial to this Convention, to be presented to the Legislature of Ohio, on the subject of so amending the Canal law as to permit physicians, when visiting their patients, and those calling for medical aid, to ride on the tow-path. Whereupon, Doctors Gard, Thompson, and Ewing, were appointed said committee.

On motion of Dr. Thompson,

*Resolved,* That Dr. T. D. Mitchell be requested to deliver a discourse upon the subject of a Lunatic Asylum, on Thursday evening next, at 8 o'clock, and that the citizens of Columbus, both Houses of the Legislature, and strangers now in the city, be respectfully invited to attend.

The Chair appointed Doctors Awl, Parsons and Wright, a Committee of Arrangements, to report the time and place of the delivery of the addresses of Doctors Drake and Mitchell.

Dr. DRAKE, from the committee on the subject of a School for the Education of the Blind, made the following

### REPORT:

The Committee to whom was referred the subject of a School for the Education of the Blind, to be established by the State of Ohio, near her Capitol, beg leave to offer the following REPORT:

From the best data which the committee have been able to obtain, there are in the State, at least 500 blind persons; that is, one for every 2,000 inhabitants. It is impossible for these unfortunate individuals to participate in any of the ordinary means and modes of instruction, except those which prevail in the families to which they belong, and which, it is well known, are entirely inadequate. Your committee, therefore, are decidedly of opinion, that it is the duty of the State to create an institution, where the permanently blind may receive that instruction which will enable them to participate more extensively in the enjoyments of society; provide for their own support, and fit themselves for a state of happy future existence.

The methods of instructing the blind in literature, are peculiar and expensive; and a public establishment, with qualified teachers, well provided with appropriate books and apparatus, is indispensable. Having such aids and facilities, it has been demonstrated, the blind may learn spelling, reading, composition, writing, arithmetic, and geography; indeed, make themselves able proficients in every branch of literature and science, as rapidly as children and young persons, of equal native intellect, who are not deprived of their sight.

It is equally certain moreover, that they may be taught a great variety of useful mechanic arts, such as making baskets, mats, cordage, and nets; bottoming chairs, spinning, sewing, plaiting straw, and weaving; and even become good house joiners, book binders, and shoe makers, not to extend the catalogue of employments.

They may, also, be taught to perform on musical instruments of every kind, in a scientific manner; and, as in general they possess much native taste and talent for music, be thereby in many instances enabled to earn for themselves a support, while they add not a little to their enjoyments.

In the opinion of the committee, these views only, should induce the General Assembly to extend its fostering arm over these children of misfortune; but there is a different aspect in which the subject may be presented. From the difficulty under which the poorer classes of society do now and must forever labor, in obtaining early and efficient medical aid, a large proportion of the blind in every community, belong to that order in society. Many of these bereaved and pitiable members of the community are, therefore, perpetually sinking into pauperism, and becoming permanent charges upon the townships, while if they were taught some profitable mechanical occupation, they would be able to support themselves.

There are other considerations still, that cannot fail to press themselves on the mind of the General Assembly, as they have on that of your committee.

First. It is well known, that in disposing of the public lands, Congress have reserved and put under the management of the State, a thirty-sixth part, for the support of popular education, besides three entire townships, for the endowment of colleges and universities.

Second. The State of Ohio has instituted a system of free schools, to be supported by a property tax.

Now the blind, at the present time, enjoy no participation in these public provisions, although in *equity* they are entitled to an equal, in *charity* to a larger, share, than other children; and hence their claims rest on the broad foundations of justice and humanity. Under this view of the subject, your committee are of opinion, that the Convention should earnestly but respectfully press upon the General Assembly, the duty of erecting, furnishing and endowing, in or near the capital of our State, an institution for the instruction, in letters and the mechanic arts, of this unfortunate portion of our population.

For this purpose a direct appropriation from the treasury of the State, of that which, is annually due to the blind, but never expended for them, has been accumulating from its first settlement, should immediately be made. Of the sum necessary, your committee do not feel it their duty to speak; but they may say, that it need not at first be great; for the number of pupils at the beginning would be small, and an edifice, suitably planned, might be enlarged, according to the wants of the public. Your committee would also suggest, that such appropriations might be prospective, and made on the condition that a certain additional sum for the same object, should be contributed by individual beneficence.

As to the annual support of the institution, your committee would observe: 1st. That those who are able to pay, should be charged a price sufficient to cover all their expenses, and contribute something, moreover, to the salaries of the teachers. 2d. That it should be made obligatory on the Trustees or Overseers of the Poor, of the different townships, to send to the institution, for a term of years, at their own expense, all the blind children and young persons, who are paupers, at a price that should cover their expenses. 3d. That to make up the deficiency, an adequate *per centum* of the revenues arising from the school lands granted by the General Government, and from the tax whereby common schools are supported, should be directed upon this object.

Your committee will only add, that schools for the education of the blind have recently been instituted in Boston, New York, and Philadelphia; that in the short time they have been in operation, they have produced results at once consolatory and cheering to humanity; and that one of their most distinguished superintendants, has written to the person who has the honor to draft this report, that should the State of Ohio undertake the establishment of such a school, he will, if his duties at home can be so arranged as to permit him to be absent, cheerfully devote several months to its organization; without any other compensation, than the reimbursement of his current expenses.

In conclusion, your committee would respectfully express the hope, that every member of the Convention, will regard it as a duty to his profession, to enlighten the public mind and warm the hearts of his friends and neighbors, on this deeply interesting subject. In proportion as physicians shall labor to institute or advance public works of charity or patriotism connected with the medical profession, it will assume a higher rank in society, and acquire that dignity and moral distinction, which it may justly claim.

Your committee beg leave to offer the following resolutions:

1. *Resolved*, That a manuscript copy of this report be transmitted to His Excellency the Governor, with the request that he will lay it before the General Assembly.

2. *Resolved*, That 500 copies be printed for distribution, by the members of the Convention, among the people of Ohio.

3. *Resolved*, That it be published in the newspapers of Columbus.

All of which is respectfully submitted.

DAN. DRAKE, *Chairman*.

*Jan.* 6, 1835.

Which Report was adopted.

On motion of Dr. Reed,

*Resolved*, That a committee of three be appointed by this Convention, to inquire into and report upon the expediency of forming a State and other independent Medical Societies. Whereupon, Doctors Reed, Parsons, and Wright, were appointed said committee.

Dr. Wetmore was excused from serving on the Temperance Committee, and the chair appointed Dr. Drake in his place; who, on behalf of the committee, reported, *instanter*, the following resolution, which was unanimously adopted:

*Resolved*, That in the opinion of this Convention, it is in the power of the members of the medical profession, to exert a great and most salutary influence on the community, against the use of ardent spirits, and that it should be regarded as a professional duty, to labor in every convenient mode to effect that object.

Whereupon, the Convention adjourned to meet at this place to-morrow, 9 o'clock, A. M.

*Wednesday Morning, Jan.* 7, 1835—9 *o'clock.*

The Convention met pursuant to adjournment. The President in the chair. The minutes of yesterday were read and approved.

Dr. Coleman, from the committee on the subject of Vaccination, made a Report, which, on motion, was ordered to lie on the table.

Dr. Kreider, from the committee on the subject of memorializing the Legislature on the propriety of legalizing the study of Anatomy, made the following

### REPORT:

Your committee, appointed to report upon the propriety of memorializing the Legislature on the subject of passing a Law to authorize and permit the dissection of dead human bodies, for the purpose of acquiring a practical knowledge of Anatomy, have considered thereof, and now REPORT:

That in their opinion, the enactment of such a law would be highly beneficial to the cause of humanity, and is loudly called for by the best interests of human society. All must admit that the knowledge of that science, acquired at our colleges and schools, is of a character that in its details and practical benefits, is soon, at least partially forgotten. It is necessary to the practitioner of surgery, however conversant he may have been with human anatomy in early life, that he should perform occasional dissections to revive his knowledge of the structure of the human system, so that he can, without embarrassment, perform many of the capital operations of surgery.

How can this be accomplished so long as a statute exists rendering it *penal* to procure a body for dissection. So long as such a law is in force, so long will it be a strong bulwark around the long established and common prejudice that prevails among the mass of mankind against dissections. Let the law be repealed, and you take away much of the vantage ground of its opposers. And why should not those persons, who, by their crimes, have rendered themselves a nuisance and an expense upon community, and have thus become the property of the country, at their death become subjects for the use and benefit of the country that has been burdened with them? All must admit that dissections *must* be practised somewhere and in some manner, else how can any knowledge of the science be attained? Is it not, then, more just, more politic, more consistent with equity, that this class of individuals should be selected, rather than any other? Surely it is. Impressed with the truth of this proposition, your committee offer for adoption the following Resolutions, to wit:

1. *Resolved*, That this Convention deem it expedient and proper, to memorialize the Legislature to so amend the criminal law, as to modify or repeal the 19th section of the act entitled "An act for the punishment of certain offences therein named," passed March 8th, 1831; and further praying the enactment of a law authorizing the dissection of all persons convicted of capital offences, who die either in common jails or the Ohio penitentiary, or that are hanged in pursuance of law.

2d. *Resolved*, That a committee of three members be appointed, to draft a memorial in the name of this Convention, andpresent the same to the Leg slature at its present session, in accordance with the foregoing views.

Respectfully submitted,

M. Z. KREIDER, *Chairman.*

Which Report was adopted, and Doctors Johnson, Kirtland, and Parsons, were thereupon appointed to prepare and present such a memorial to the Legislature.

On motion of Dr. Cooper,

*Resolved*, That a committee of three be appointed, to inquire into the probable cost of publishing the journal and proceedings of this Convention, and that the quota of each member of the Convention be assessed and collected as speedily as possible. Whereupon, Doctors Cooper, M'Niell, and Reed, were appointed said committee, who thereupon reported, as follows, to wit:

Your committee, appointed to ascertain the probable cost of printing the journal and proceedings of this Convention, and for the purpose of assessing upon each member his proper quota, REPORT:

That from the best information they have on the subject, the expense accruing to the Convention will be about seventy dollars, and that the sum of one dollar and fifty cents assessed upon each member, will, in all probability, cover all expenses; and that this calculation contemplates the publication of 1000 copies of the Journal and Proceedings of this Convention, and 500 copies extra of Dr. Drake's Reports upon the subjects of Commercial Hospitals, and a School for the Blind. Which Report was agreed to.

On motion of Dr. Thompson,

*Resolved,* That this Convention recommend to the medical profession in Ohio, triennial Conventions, to be held in this city.

Dr. Awl, from the committee on the subject of a Medical Journal, made the following Report, to wit:

The committeee appointed to report on the establishment of a Medical Journal under the sanction of this Convention, REPORT:

That they regard the full success of such an enterprize extremely doubtful; they therefore recommend that the project for the present be abandoned. Which Report was agreed to.

Dr. Wright, from the committee on the subject of the Leech, reported, as follows, to wit:

The committee to whom was referred that part of the medical circular which relates to a convenient supply of the Leech, have had the same under consideration, and as they are unable to make any useful suggestions upon the subject, ask to be discharged from its further consideration. Which Report was agreed to, and the committee discharged.

Dr Reed, from the committee on Independent Medical Societies, made the following

### REPORT:

Your committee, after due reflection upon the subject of "*Independent Medical Societies,*" have become satisfied that very little presents itself, which requires the action of this Convention.

They deem it proper to remark, however, that the voluntary organization of such Societies, in the large towns and populous counties of the State, would, in their opinion, prove highly conducive to the mutual improvement of their members and the elevation of the profession in general, by the spirited emulation and the high and honorable tone of feeling which such associations rarely fail to produce.

Men of the same profession should consider the assembling of themselves together, occasionally, as a duty they owe, not only to themselves, but to their profession and to the community. By this means serious misunderstandings are frequently prevented or annihilated—the social affections are cultivated—noble resolves are entered into—and an interest is soon thrown around the meetings; arising either from recollections of the past, or from the opportunities which may offer for present or future action in the cause of philanthropy and science, well calculated to elevate the feelings, and strengthen the love of knowledge, which are so truly desirable in the character of a physician.

1st. *Therefore, Resolved,* That this Convention recommend to the physicians of Ohio, the formation of *Independent Medical Societies,* for the purpose of mutual improvement and the rearing of that high standard of professional honor and etiquette, which is of much individual advantage, and cannot fail to elevate the medical character of the State.

2d. *Resolved,* That this Convention recommend *triennial* conventions of the physicians of Ohio, who feel an interest in the organization, advancement and elevation of the medical profession, as well as the promotion of objects of general benevolence. S. REED, *Chairman.*

Which Report was agreed to.

Dr. Reed, from the committee on the subject of Medical Ethics, made a Report, which recommended to the physicians of Ohio the able work of Dr. Percival, as the standard of professional etiquette; when, on motion of Dr. Drake, said Report was recommitted to the same committee, with instructions to prepare a digested code of Medical Ethics, *adapted to the state of the profession of Ohio.* Dr. Kirtland, a member of said committee, was then, by his request, excused from serving further on the same, and Dr. McNiell was appointed in his stead.

On motion of Dr. Reed,

*Resolved,* That this Convention appoint a committee of three, to make the proper arrangements for, and superintend the publication of its proceedings—to be done in the best and cheapest manner. Committee, Doctors Reed, Rives, and Parsons.

Dr. Gard, from the committee on the subject of the Canal Law, read a memorial to be presented to the Legislature, on the amendment of the Canal Law. Which Report was ordered to lie on the table.

Dr. Rives, from the committee on the subject of Medical Education in Ohio, made the following

### REPORT:

The committee appointed to inquire into and report the means of improving the state of Medical Education in Ohio, have given to the subject all the consideration which their time and other duties connected with the business and proceedings of this Convention would allow. Ample observation has convinced your committee of the existence of radical defects in our systems of education; defects which in many instances have exerted a disastrous influence upon individual fortune, and if not corrected, must ultimately annihilate whatever of respectability remains to the profession.

To arrest this proclivity of medical character, is the duty of every physician. All cannot exert an equal influence in this hallowed work, but there is not one of us who may not contribute, by his instructions or his example, to its success.

It appears to your committee, that of all the means which could be devised for the elevation of the medical profession in the State of Ohio, the adoption and recommendation by your honorable body, of a judicious preparatory and professional course of studies would be most effective. They are aware that this standard cannot now be raised so high as it is in Europe, or as they could desire to see it; but they are nevertheless persuaded, that some standard, accommodated to the condition of our State, which shall exclude the imbecile and illiterate, who are annually assigned to the ranks of the profession, by vanity, or indolence, or some worse motive, may be adopted.

Under a full persuasion of the correctness of these views, your committee will proceed at once to unfold their opinions in relation to the qualifications and preparatory education of students of medicine, the duties of private instructors, and the objects and organization of medical schools.

1st. *Of the qualifications and preparatory education of Students of Medicine.*—There is perhaps no human pursuit which requires a more varied combination of physical, moral, and intellectual qualities for its successful study or prosecution, than the profession of medicine; and yet there is none in which considerations of fitness are more completely disregarded.

Your committee would recommend, among the natural endowments which should characterize the student of medicine, a sound constitution and a vigorous mind, *sana mens in sano corpore*, should be an indispensable pre-requisite. Without health, it is impossible to prosecute successfully, the study of either of the learned professions. Close application and sedentary habits induce a train of unpleasant symptoms in the most robust individuals, but in the delicate and predisposed, they never fail, if long protracted, to produce fatal maladies. But if physical developement be necessary as a means of securing the operations of mind, and in this manner constitute a pre-requisite to the study of medicine, the character of the *intellect itself* must form a much more important consideration. Observation and judgment are the most valuable faculties which a medical student can possess. By their aid he is enabled to comprehend the principles of the science, and to apply them to the treatment of diseases. If to these faculties he add inquisitiveness and ambition to impel him forward in the acquisition of knowledge, industry to sustain him, and a gentlemanly demeanor, the offspring of a benevolent and feeling heart, his intellectual and moral qualifications may be considered as complete. But education has yet to impress her seal upon the character of the individual, before he can be regarded as suitably prepared for the study of medicine.

In relation to many of the branches of learning taught in our Universities, their importance is variously estimated by different individuals. No diversity of opinion, however, it is presumed, can exist as to the indispensable necessity of a correct elementary education, including grammar, the art of composition, physical geography, and the general outlines of history. To these should be added, if the student aspire (which he should always do) to the elevated places in the profession, a respectable attainment in classical learning, in mathematics, and the natural sciences. Chemistry is so generally recognized as a valuable acquisition to the physician, that it is already adopted as a branch of study in every medical school. Natural philosophy, botany, zoology, and mineralogy are less generally admitted among the preparatory studies, but their relations to medicine are sufficiently obvious to make them objects of interest to the inquisitive and enlightened student. Without some knowledge of mechanical laws, we should be unable to understand many of the functions of the human system, such as seeing, hearing, respiration, and circulation. Botany and zoology furnish important aids in our physiological researches, but the former, as does mineralogy, connects itself immediately with the materia medica.

Your committee have indicated the branches of learning which they would recommend as a preparation for the study of medicine, without any attempt, by elaborate exposition, to show the relation of its several parts to our science. Without pretending to attach equal value to every part of the scheme, your committee would take leave of the subject with the expression of an ardent hope, that the members of this Convention will all unite their efforts in favor of a more elevated scale of preparatory and auxiliary studies, and thus throw their individual influence in the cause of regenerating the profession.

The *second* branch of the enquiry which we proposed in the investigation of this subject, related to *private pupilage*. Of the numerous defects of this system, and their deleterious operation upon the profession, it would be vain to attempt an account. We shall be content to draw your attention to a few of the most obvious duties of private preceptors.

Whenever an individual makes application to a physician for admission into his office as a pupil, it will be his duty to examine the candidate rigidly as to his preparation. If he discover any deficiency in the essential branches of a proper preliminary education, it is the duty of the physician to admonish him of his incapacity, and to advise him to apply himself to some other avocation. But if his examination be approved, a more pleasant duty awaits the physician. The candidate is to be apprized of the *magnitude* of the undertaking which he has assumed, and of the importance of diligence and method in his studies. For the purpose of securing these results, it will be the duty of the preceptor to institute regular and systematic examinations. By these means the mind of the pupil will be deeply impressed with the subjects of his reading, and he will be stimulated to industry and exertion. But it is not the duty of the preceptor to aid his pupil by his prelections and examinations *only.* He must provide his office with plates, anatomical preparations, and all other facilities which can assist the progress of his pupils. By the assiduous and faithful discharge of these duties, he will have the satisfaction of seeing his pupils daily improving in knowledge, and ultimately prepared to take an honorable position in the ranks of the profession.

There are two defects in private pupilage, connecting themselves with the student, which, in the opinion of the committee, deserve the serious consideration of medical men.

The first is the period of pupilage. This is much too short. It is not at all unusual for the student to commence the practice of medicine at the end of eighteen months or two years. The influence of the profession should be brought to bear upon this evil until it is rectified, and medical character is redeemed from the reproach of incompetence, thus brought upon it.

The second defect to which allusion was made, is the encouragement held out to men of slender means, to engage in the study of medicine, when it is almost certain that their pecuniary situation, must compel them either to abandon the pursuit, or to engage in it prematurely and without preparation—a course of this kind is cruel to the individual, and a crying sin against the interests of humanity.

3d. *Medical Colleges.*—These are important institutions. They are designed to perfect and complete medical education, which generally commences under private preceptors. It is evident from this consideration, as well as from the influence they radiate through the country in which they are located, that much care and attention should be bestowed on their organization. The following propositions in relation to this subject, will be generally assented to:

1. The professorships should be filled with men most eminent for abilities and attainments, who should combine with these qualifications an aptitude for teaching.

II. *The number of professors.*—On this subject some diversity of opinion prevails. Six, seven, and eight have been advised. Circumstances may occur to render the one or the other proper. But your committee entertain the opinion, that where no peculiar cause operates to affect the decision, the number sholud correspond to the natural division of medicine, into 1st, the Institutes of Medicine. 2d, the Theory and Practice of Medicine. 3d, Materia Medica. 4th, Anatomy. 5th, Institutes of Surgery and Operative Surgery. 6th, Obstetrics and the Diseases of Women and Children. 7th, Chemistry. Medical Jurisprudence to be distributed according to its affinities.

III. Experiments and demonstrations should constitute a principal object in medical schools. Much that is taught in lectures might be as successfully studied in the closet; but chemistry, which is an experimental science, anatomy, operative surgery, and obstetrics, which are demonstrative, can no where be so advantageously cultivated, as in public institutions, where the means of illustration are employed by the professor, and may be repeated by the student.

For the sake of these facilities, as well as for many other reasons, every individual who aspires to become a *useful* physician, should feel himself under the highest obligations to attend lectures, if not to graduate, in some respectable medical college. Unless he do this, his education must be regarded as radically defective, and his practice cannot fail to conform in its general character, to his education.

In the opinion of your committee, much advantage would result to the cause of medical education, if by a combined and associated action between the medical institutions of our country, their sessions could be prolonged, and the standard of professional attainment elevated. Your committee are aware of the difficulty, of the impracticability, indeed, of an independent movement in these public matters; but they feel that the interests and dignity of the medical profession are so deeply concerned in these reformations, that they have assumed the responsibility of throwing out these hints, under the hope that at some future time they may be acted upon by those who only are competent to afford relief in the premises.

L. C. RIVES, *Chairman*.

*Jan.* 7, 1835.

Which Report was agreed to.

Dr. Thompson, from the committee on that subject, Reported as follows on the subject of a Lunatic Asylum:

### REPORT.

Humanity and the character of the State of Ohio, call imperiously for the erection of an Asylum that will be creditable to the State, and in all respects adapted to the relief and care of mental derangement. On this topic, it is believed that no diversity of opinion obtains in the medical profession; and under these impressions, the committee beg leave to submit for your consideration, the following memorial to the Legislature now in session.

*To the Honorable, the Legislature of Ohio,*
*In General Assembly met:*

The Medical Convention assembled in this city, deeply impressed with the importance and absolute necessity of having a suitable establishment for the treatment of insane persons, most respectfully submit to your honorable body the following facts and considerations in the premises.

It is known to the Legislature that an institution exists in the city of Cincinnati, bearing the appellation of a Lunatic Asylum; and it is equally well known that it has proved totally inadequate to the ends for which it was designed, being in fact nothing more than a prison. The Faculty of the Medical College of Ohio, who are, by law, the attendants on that establishment, have repeatedly declared their conviction that it is wholly in vain to attempt the restoration of reason, of those whom disease has rendered the unhappy subjects of confinement therein, and that the arrangement throughout is rather calculated to render insanity permanent, than to promise a cure, or even a partial restoration. This being the case, no member of our profession can advise that the insane in his vicinity be sent to Cincinnati, and consequently Asylums in other States are recommended.

Your memorialists need scarcely remark to your enlightened body, that the time has already gone by in which the ideas of a *prison* and Lunatic Asylum were inseparably associated in the mind; and thrice happy is it for the *unfortunate insane*, that the progress of science and the enlightened benevolence of the age, are fast dispelling the remnant of barbarism. so revolting to humanity.

That, under proper circumstances, insanity in most cases is curable, is a fact too well established to admit of a doubt. This then, in connection with the fact that of all God's creatures on earth, none are so awfully calculated to excite the moving sympathies of our nature to deep commisseration, as is the form of man deprived of reason, should, and it is believed will, enlist in behalf of the scheme of your memorialists, the favorable consideration of the christian, the patriot, and the philosopher.

The deaf mute, though deprived of a portion of the good gifts of a bountiful Creator, is in many respects a reasonable and intelligent creature, and susceptible of a high degree of moral and intellectual culture. The same remark will apply to the unfortunate blind. They both demand of savage and civilzed man a portion of sympathetic pity; nor is there in existence a heart so cold as to be unwilling to concede the justice of such a demand. The deaf and the blind are, we say, rational creatures, both calculated to impart to their friends the means of enjoyment, and in return, to experience many of those feelings of affection and comforts of existence, which render life so desirable to the family of man. Indeed, so true is this remark, that we often see the affections of a family concentrated upon such unfortunate member, even to favoritism; and this must in some degree compensate for the privation of which the individual is the subject. But not so with him who is insane. Himself an object of heart-rending commisseration, he is equally incapable of receiving or imparting joy. View the subjects of every variety of insaniiy, from the drivelling idiot to the furious maniac, and what is to be seen but objects of pity, which, when disconnected with the probability that they may yet be restored to the use of reason, cannot fail to rest as a leaden weight upon every feeling heart; but which, when associated with the idea of a restoration of their senses, fill every heart with benevolent resolves.

Your memorialists, therefore, only deem it necessary to call your attention particularly to the fact that has been already stated, (the entire unfitness of the present establishment bearing the name of Lunatic Asylum,) in conjunction with the fact that there is now in your State, not less than 600 to 1,000 insane persons, entirely destitute of the proper means of recovery, to ensure such legislation as the pressing importance of the subject demands.

In a State which has already expended her millions in the construction of commercial highways and literary and benevolent institutions—in a State wealthy in her resources and proud of her wealth, a call from the unfortunate cannot be heard in vain.

In regard to the location of the Asylum, your memorialists are of opinion, that a situation more central than Cincinnati should be selected. In such a project the convenience of every part of the State should be consulted, as all have an equal interest therein. In this view of the subject, it would seem that no place presents so many advantages as the city of Columbus, and it is believed that none would be more acceptable to the community at large.

The Medical Convention would not so far infringe upon the rights and privileges of your honorable body, as to be wiliing to make any suggestions in relation to the funds proper to be called into requisition in the erection of the establishment contemplated in the memorial, nor as to the means of supporting the same, but would respectfully express it as their opinion, that to be useful in the highest degree, there should be attached to it a sufficient quantity of ground to answer the purpose of gardening and such other labors and amusements as the nature and objects of such an institution might require; and would also beg leave further to recommend to the notice of the Legislature, the "Friends' Asylum," near Philadelphia, and a similar institution at Hartford, Connecticut, as suitable models for the guidance of those to whom the erection of the contemplated Asylum may be committed; and for much information upon the subject at large, to the report of the "Board of Commissioners appointed by the General Assembly of Ohio, March the first, 1831, to visit the Commercial Hospital and Lunatic Asylum of Ohio, at Cincinnati." And your memorialists, as in duty bound, will ever pray.

R. Thompson,

Thos. D. Mitchell,

Wm. M. Awl,

Jno. Eberle,

Edwin W. Smith.

} *Committte.*

[After the reading of the above Report, Dr. Drake offered a resolution, declaring that the present Lunatic Asylum at Cincinnati is inadequate to the wants of the State, and in all respects imperfect and requiring improvement, but omitting a recommendation to remove it to Columbus. The chair decided that the resolution was out of order.

The manuscript has not come into the hands of the publishing committee, who design by this note to supply the omission.]

Which Report was agreed to, and on motion, the memorial was ordered to be signed by the officers and forwarded to the General Assembly.

On motion of Doctor Kreider.

*Resolved*, That the digested code of Medical Ethics published in the proceedings of the General Medical Society of Ohio, in the year 1831, be published in the proceedings of this Convention, viz :

Rule 1. It is the duty of every medical practitioner to treat his patients with steadiness, tenderness and humanity, and to make due allowances for that mental weakness which usually accompanies bodily disease. Secrecy and delicacy should be strictly observed in all cases in which they may seem to be peculiarly required.

2. The strictest observance of temperance cannot be too strongly inculcated on the minds of the practitioners of medicine and surgery, a clear and vigorous intellect and a steady hand, being absolutely necessary to the successful practice of those branches of medical science.

3. Unfavorable prognostication should never be made in the presence of patients; yet, should there seem to be immediate danger, it becomes the duty of the medical attendant to apprise the patient's friends of that circumstance.

4. In every instance in which one physician has been called on to visit the patient of another, a consultation with the the former medical attendant should be proposed. Consultation in difficult cases should always be recommended, and the physician called on for that purpose, should always pay the greatest degree of respect to the practitioner first employed, and allow him the privilege of delivering all the directions agreed upon.

5. Special consultations are sometimes wished for; in such cases the physician called on should carefully guard against paying another visit, unless he should be requested to continue his services by the patient, or some of his friends.

6. When one physician is called on to visit the patient of another in his absence, or during short indisposition, he should not manifest a wish to continue in attendance any longer than the physician first called on should be able to resume the charge of the case, unless a continuance of his services should be expressly wished for by the patient or his friends.

7. Physicians should not visit their patients too frequently, lest seeing them oftener than necessary might produce unsteadiness in the treatment.

8. Theoretical discussions should not be too freely indulged in consultations, as they frequently give rise to much perplexity, without any improvement in practice.

9. The junior physician in attendance should always deliver his opinion first, the others according to seniority, and a majority should decide; but in the event of a tie, the physician first in attendance should give the casting vote in regard to the future treatment, and to him should be entrusted the future management of the case, unless the patient or his relations should object to his being continued.

10. Although the possession of a diploma, honorably acquired, furnishes presumptive evidence of professional ability, and entitles its possessor to pre-eminence in the profession, yet, the want of it should not exclude practitioners of experience and sound judgment from the fellowship and respect of the regular graduate.

11. In consultations, punctuality in meeting at the same time shoul be strictly observed, but the physician who first arrives, should wait for a reasonable length of time for the arrival of others. A minute examination of the patient, however, should not take place, until one or more of the medical attendants are present, except in cases of emergency. All subsequent visits should, if practicable, be made by mutual agreement, and no medical discussion should take place in the presence of the patient.

12. Attendance on members of the profession or their families, should always be gratuitous, but should not be officiously obtruded. Should the circumstances of the medical practitioner indisposed, enable him to make a recompense for medical services rendered to himself, his wife or family, it is his duty to do so, especially if he reside at a distance.

13. When one medical practitioner is called on to visit a patient whose recovery has been despaired of by the physician first in attendance, and the disease should afterwards terminate fatally under his management, he should avoid insinuating to the friends of the deceaseds that if he had been called on a day or a few hours sooner, he could have effected a cure. Such a course of conduct is highly reprehen, sible, and empirical in the extreme. And, in the event of the patient'- recovery, such a person should not assume all the credit, as the cure might have been partly effected by the medicines prescribed before he took charge of the case.

14. The use of nostrums and quack medicines should be discouraged by the faculty, as degrading to the profession, injurious to health, and often destructive of life. Should patients laboring under chronic complaints obstinately determine to have recourse to them, a reasonable degree of indulgence should be allowed to their credulity by the physician; but it is his sacred duty to warn them of the fallacy of their expectations, and the danger of the experiment, and the necessity of strict attention to the effect produced by them, in order that their bad effects, if any, should be timely obviated.

15. No physician should, either by precept or example, contribute to the circulation of a secret nostrum, whether it be his own invention, exclusive property, or that of another. For, if it be of real value, its concealment is inconsistent with beneficence and professional liberty, and if mystery alone give it value and importance, such craft implies either disgraceful ignorance or fraudulent avarice.

16. In all cases where diversity of opinion and opposition of interest give rise to controversy or contention between two or more members of the profession, the decision should be referred to a sufficient number of physicians, as they are frequently the only persons in the community capable of properly estimating the merits of the dispute. But neither the subject litigated, nor the decision thereon, should be communicated to the public, as individual reputation might suffer, and the credit of the profession generally be injured.

17. A wealthy physician, or one retired from practice, should refuse to give gratuitous advice, unless the danger of the case (the absence of the practising physician) or the poverty of the patient should warrant him in so doing. In all cases where he may be preferred, he should recommend a consultation with some one engaged in active practice. This rule should be strictly observed, as a contrary course is gratuitously depriving active industry of its proper reward.

18. When a physician is called on suddenly to visit the patient of another, in consequence of some unexpected or alarming change in the symptoms, he should adopt a temporary plan of treatment, suited to present circumstances. He is not warranted in interfering afterwards, unless requested to take charge of the case, when he should propose an immediate consultation with the physician previously employed.

19. Physicians should never neglect an opportunity of fortifying and promoting the good resolutions of patients suffering under the bad effects of intemperate lives and vicious conduct; and, in order that their counsels and remonstrances may have due weight, it will readily be seen, that they should have full claim to the blameless life and high moral character which we have stated to be a necessary pre-requisite to an honorable stand in the profession.

20. Medical men should "remember the Sabbath day to keep it holy;" and visits should, as far as consistent with professional engagements, be made either before or after public worship, or during its intervals.

On motion of Dr. Drake,

"*Resolved*, That in the opinion of this Convention, it would greatly promote the practice of Vaccination, for the General Assembly to enact a law, requiring, That after a limited time no child shall be admitted into the public schools, who has not been previously vaccinated."

On motion of Dr. Drake,

*Resolved*, That the thanks of this Convention be returned to the Marshal of Ohio, for the use of the room in which we have met, and also to the trustees of the Presbyterian church, for the use of the room in which this Convention was organized.

On motion of Dr. Earl,

*Resolved*, That the thanks of this Convention are due to the officers of this Convention, for the able and impartial manner in which they have discharged the duties of their respective stations.

On motion of Dr. Drake,

*Resolved*, That after having visited the State Asylum for the Deaf and Dumb, the members of this Convention feel it a duty to express the most decided approbation of its plan, arrangements and discipline, not less than of the progress of improvement in the pupils.

On motion of Dr. Cooper.

*Resolved*, That the thanks of this Convention be tendered to Dr. Wm. M. Awl, for the very efficient part he has taken in originating this Convention, and the prompt attention he has shown to the comforts of this Convention as a body.

On motion of Dr. Drake,

*Resolved*, That after having visited and inspected the Penitentiary of the State, the members of this Convention highly approve of the discipline and police of that establishment.

On motion of Dr. Drake,

*Resolved*, That it shall be the duty of the Secretaries of this Convention, to give at least three months' notice, in the newspapers of this city, of the next meeting of the Convention.

On motion,

The Convention adjourned until the first Monday of January, A. D. 1838.

[Attest] PETER ALLEN, *Pres't.*

M. Z. Kreider, *Rec. Sec'y.*

Wm. M. Awl, *Cor. Sec'y.*

